# Using learning curves to guide the energy transition with the example of heavy electric trucks

**DOI:** 10.1038/s44333-025-00029-5

**Published:** 2025-04-02

**Authors:** Auke Hoekstra, Floor Alkemade

**Affiliations:** 1 Department of Mechanical Engineering, Eindhoven University of Technology, Eindhoven, The Netherlands; 2 Zenmo simulations, Heusden, The Netherlands; 3 Department of Industrial Engineering and Innovation Sciences, Eindhoven University of Technology, Eindhoven, The Netherlands

**Keywords:** Environmental impact, Energy efficiency, Energy policy, Energy supply and demand, Energy and behaviour, Electrical and electronic engineering, Energy grids and networks, Mechanical engineering, Energy grids and networks

## Abstract

Learning curves accurately predict the continuing progress of clean energy and mobility technologies but are not systematically used as a basis for evidence-based policy. We present a systems-level learning model for electric trucks to illustrate how this can be done. Focussing on Europe, we use an approach based on learning curves for eTruck drivetrain and battery pack design; battery developments in cost, durability and composition; energy efficiency and CO_2_ emissions; weights of all components; electricity and diesel costs; charging costs in different scenarios; and the use of an eTruck fleet with different ranges. Our model shows several tipping points that can lead to fast eTruck adoption. Policies could leverage these tipping points by rewarding longer range, faster charging, vehicle-to-grid capabilities, and an open and interoperable network of eTruck fast-chargers to drive a rapid and cost-effective transition to eTrucks.

## Introduction

Analysing the data for energy transition technologies like solar, wind, batteries and electric vehicles reveals learning curves that imply large and speedy cost reductions and performance improvements. In order to accelerate the energy transition, the IPCC calls for government strategies that simultaneously weaken high-carbon systems and stimulate low-carbon alternatives^1^. Leveraging learning curves is a way to do that. Despite the accuracy of learning curves in predicting progress, they are underused as a basis for policy. Using the example of battery electric heavy trucks (eTrucks), this paper demonstrates how a systematic approach that makes extrapolating learning curves the default, leads to better predictions that could accelerate the energy transition and ramp up timely policy support.

Heavy trucks cause over a quarter of greenhouse gas road emissions^2^ and this percentage is growing^3^. ETrucks produced with and running on renewable energy could reduce these emissions to almost zero. Since 2017, there has been an increasing body of research indicating that eTrucks will get a decisively lower total cost of ownership than alternatives^4–9^. In the cost sensitive market of trucking, this implies they could quickly take over.

However, there is a persistent misconception that electrification is somehow harder for heavy trucks than for cars, leading policymakers to hedge their bets and drag their feet. For example, the ambitious “road to zero” initiative^10^ avoids singling out electrification as the preferred alternative for heavy trucks and does not commit to choices that would accelerate the adoption of eTrucks. Similarly, the current blueprint for transportation carbonisation of the United States^11^ still claims that short-haul eTrucks are less of an opportunity than electric cars and that long-haul trucks will primarily run on hydrogen, efuel and biofuel, even long term. These policies do not only neglect the recent developments on the cost-effectiveness of eTrucks (like the fast reduction in battery prices), but they also create policy uncertainty. This policy uncertainty, in turn, deters private investment in eTrucks and eTruck infrastructure.

While the accuracy of using learning curves for predictions has been demonstrated^12^, they are typically applied at the component level. This paper argues that an integrated systems approach improves accuracy and usability of learning curve evidence. It looks not only at multiple eTruck components, but also at changes in eTruck designs, cost per ton kilometre, and fleet composition. This integral approach shows that cost parity could be achieved relatively quickly, especially once supporting policies that e.g., lead to charging corridors are in place. All in all an integral learning curve approach can help to provide the clarity that policy makers need before they commit to strong support policies.

This paper builds upon but goes beyond the recent eTruck literature by making the learning curve approach and its implications more explicit. It first demonstrates the use of learning curves as an evidence base for policymaking by re-analysing recent expert forecasts on batteries and eTrucks. We then outline a systematic approach to use learning curves to create accurate forecasts. Like Kaur et al.^13^ we use cost per ton kilometre (tkm) in combination with detailed weight calculations. We include batteries and the lighter electric drivetrain^14^ but also the implications of next-generation truck designs with multiple motors and structural batteries. This gives insight into the continuously decreasing weight disadvantage of eTrucks and makes it endogenous to the cost calculation.

Finally, we use fleet composition when comparing the cost of eTrucks against alternatives. We show how a fleet with eTrucks that can drive different ranges allows the fleet operator to fulfil all trips at significantly reduced cost per tkm.

## Methods

### Using learning curves as the default approach

**Fig. 1:**
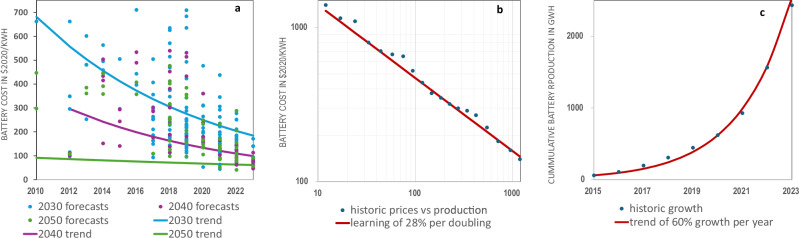
Learning curves best predict battery prices. Forecasts for battery prices are constantly lowered, in a way that follows an extrapolation of historic learning. **a** Forecasts for 2030, 2040 and 2050, ordered by year of publication. The data is from a recent meta-analysis by Link et al.^16^ who fit a trendline predicting $170 in 2030, $125 in 2040 and $100 in 2050 (*R*^2^ 0.49). We found a second correlation in the data: forecasts become lower when their year of publication is more recent. This happens in a way that conforms to the learning curve presented in panels (**b**) and (**c**) (2030 *R*^2^ 0.45, 2040 *R*^2^ 0.63, 2050 *R*^2^ 0.44). **b** Learning has been consistent at 28% for every doubling of cumulative production (*R*^2^ 0.99). **c** Battery production growth follows a logistic function that currently increases at 60% per year (*R*^2^ 1.00), which we conservatively extrapolate using the IEA STEPS scenario^17^. **b**, **c** Taken together leads to prices of $91 in 2030, $49 in 2040 and $27 in 2050.

Recent papers that show eTrucks will have a lower total cost of ownership (TCO) than diesel trucks all assume that batteries will become cheaper, some also include the effects of developments in battery weight and drivetrains, implicitly using learning curve effects. We argue that the explicit use of learning curves as a default approach will provide a stronger evidence base for such assumptions and allow comparison across studies. Currently most papers either fail to account for learning (e.g. Larson et al.^15^) or use expert forecasts that are often conservative, heterogeneous, and underestimate technological learning. A recent paper by Link et al.^16^ that provides a database of 1100 expert forecasts on eTrucks, shows that while expert forecasts are heterogeneous (best-fit curve, *R*^2^ 0.49), they do point to price reductions, reaching around $100/kWh in 2050, in line with the predictions of the IEA^17^. However, actual market developments show that pack prices already decreased to that level in 2024^18^. We advocate for a learning curve approach based on historical data. This reveals a learning curve with a 28% cost reduction for every doubling of cumulative production, which fits the observed data very closely (*R*^2^ 0.99)^12^. We also see a highly regular exponential growth of production of 60% per year (*R*^2^ 1.00)^17^. Combining both trends points to a battery pack price of $27/kWh in 2050. We reanalysed the data of Link et al. and observed that the expert forecasts are evolving with observed learning (see Fig. 1). Therefore, expert forecasts seem to follow a learning curve based on historical data instead of improving it.

Using learning curves does require a critical review of the extent to which developments can be extrapolated. At the minimum, one should check for raw material prices and make sure that exponential growth is not extended beyond what is to be expected using a logistic curve that accounts for market saturation. Moreover, a learning curve is often made up of several underlying learning curves. A well-known example is solar, where the price reduction in solar panels was partly made possible by using ever thinner layers of silicon and where overall cost analysis now focuses on installation and system cost for electricity transport and storage. Nevertheless, solar keeps growing exponentially while expert forecasts keep underestimating its growth^19,20^. For batteries, the initial reaction seems similar^21^, and while it is clear that $27 per kWh requires a switch from NMC to, e.g. LFP, Li–S, lithium–metal or sodium batteries with the liquid electrolyte preferably replaced with solid state alternatives, most of these developments are already underway. We might even see more revolutionary batteries like Li–air and fluoride shuttle batteries that could become over ten times more energy-dense than current batteries. See also Supplementary Note 1.

When using historical data to create learning curves, a key decision is where to apply these learning curves, that is, which components are expected to benefit from technological progress and cost reduction. For eTrucks, this includes the cost and weight of batteries, the cost and weight of electric drivetrains, and the cost and speed of chargers (especially if the time spent charging is a model variable). If the study also mentions CO_2_ emissions, it should include assumptions on the CO_2_ intensity of the electricity mix over the lifetime of the vehicle and of the CO_2_ intensity of production at the moment of sales^22^. Currently, studies that fit that profile seem to be scarce, although e.g. the ICCT reports are a notable exception^4,5,8,13,23,24^. In addition, the assumptions on learning should be made explicit (as in Ledna et al. Table 1)^9^ or even use a formula for yearly learning. Our main inputs are listed in Table 1^9^. More details can be found in Supplementary Note 1.

**Table 1:** Model parameters.

Parameter name	Parameter description	2022 value	Yearly learning (%)
Truck weight	Tractor excluding frame, drivetrain, and battery	4000 kg	0.5
	Tractor frame	1200 kg	0.5
	Diesel tractor: diesel drivetrain weight	2200 kg	0
	Electric tractor: electric drivetrain weight	350 kg	4
	Electric tractor: battery cells excl. pack	244 Wh/kg	4.5
	Trailer (for cargo space: pulled by the tractor)	5700 kg	0.5
Truck costs	Battery pack	$150/kWh	6.0
	ETruck powertrain	$50,000	3.0
	Diesel truck powertrain	$40,000	−1.0
Lifetime	Number of km for depreciation etc.	1,520,000 km	1.5
Energy use	diesel truck	28 l/100 km	0.7
	eTruck	1.3 kWh/km	1.0
Emissions	Truck production excl. batteries	5 kg CO_2_eq/kg	2.0
	Battery production	75 kg CO_2_eq/kWh	5.0
	Diesel emissions incl. prod.	3310 g CO2eq/l	0.4
	Fuel stack production	168 kg CO_2_eq/kW	5.0
Energy costs	Diesel excl. VAT	$1.19/l	0
	Electricity EU incl. grid and business taxes ($/kWh)	$0.10/kWh	0
	Smart charging advantage	20.0%	1.0
	Depot charging surcharge	$0.05/kWh	3.0
	Rest stop charging surcharge	$0.20/kWh	2.0
	Fast charging surcharge	$0.40/kWh	3.0

Key model parameters and their associated learning rate (see Supplementary References).

### Detailed approach and model parameters

The method used for this paper evolved over the last 10 years through discussions with electric car and trucking experts in academia and business. As described in the introduction, we developed it based on two hypotheses. First, expert forecasts are directionally correct but conservative and heterogeneous and are consistently outperformed by extrapolating observed learning curves. Second, the improvements of the eTruck business case not only come from battery price and weight, but from a host of technological developments that reinforce each other. So, the essence of our method is that we identified the most important technological developments, estimated their historical learning curve, and extrapolated that learning into the future, after checking it was plausible using expert judgement and bottom-up cost calculations. Table 1 shows the estimates that we used in our model. After we discuss the table we discuss how we determined fleet composition.

**Fig. 2:**
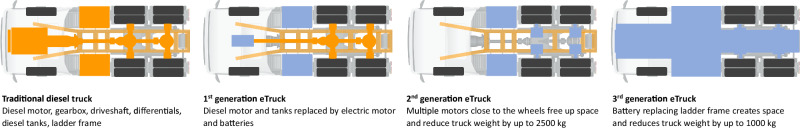
Next generation eTrucks. Next-generation eTrucks will use smaller motors and structural batteries.

Starting with eTruck weight, the drivetrain and battery are the most important. Despite the importance of drivetrain development^14,25^ and structural batteries^26–28^, only the grey literature tries to include them in the eTruck business case^5,23^. Fig. 2 shows the development we consider likely. The current 1st generation eTrucks have replaced the diesel motor with the electric one and the fuel tank with the battery. The 2nd generation will use multiple small electric motors closer to the wheels to eliminate a lot of weight currently taken for granted in diesel powertrains, such as from the driveshaft, gearbox, and differential^14,25^. This becomes possible because electric motors are tiny compared to combustion engines with the same power, need no exhaust system, and dissipate about twenty times less heat. A (fixed or two-speed) gearbox can reduce the weight of the electric motor further^25^. The 3rd generation is expected to use structural batteries^26–28^, which creates space, and reduces cost and weight.

For battery weight, we fitted learning to gravimetric cell density developments in commercially available cars and found that from 1991 to 2024, density has improved linearly with 7.36 Wh kg^−1^ y^−1^ (*R*^2^ 0.96, see Supplementary Note 1). Our model ignores battery volume because 3rd generation trucks (like the Tesla Semi) already have enough room to accommodate over 800 km of range with current batteries. We also ignore battery lifetime because we assume eTrucks will only be offered if the battery is able to outlast the eTruck and this is not an intrinsic problem^29–31^ (see also Supplementary Note 1).

Truck costs are a key part of our model since trucking is a highly competitive business with low-profit margins. In terms of CAPEX, the battery cost is the most important. In the introduction, we showed how expert forecasts are diverse and evolve over time, while learning is extremely consistent and learning curves lead to better battery price predictions vis-à-vis historical data. Predictions based on learning curves point to much lower battery prices than those based on expert forecasts. In combination with energy efficiency advancements, this leads to battery packs for a truck with 750 km of range changing from around $700k in 2010 to $100k in 2030 to $20k in 2050. A detailed step-by-step explanation, including a ‘sanity check’, is available in Supplementary Note 1. The impact of drivetrain cost is less extreme, but the eTruck starts with a $10k drivetrain cost disadvantage in 2022 and ends with a $30 advantage, which becomes more relevant for the sticker price as battery prices come down. Also, the lower maintenance costs of the eTruck drivetrain are important for the business case.

However, the biggest cost driver is the energy costs. For a diesel truck costing $100k and driving one million km, the energy costs are $300k in Europe (assuming diesel of $1.19/l or $0.12/kWh). The energy for the eTruck starts out as electricity of $0.11/kWh but quickly becomes more expensive per kWh because of the costs of grid upgrades and charging equipment (we distinguish between depot charging, overnight charging and fast charging). However, because an eTruck needs around 40% of the energy of a diesel truck per tkm (see Fig. 2b), its energy costs are around half of that of a diesel truck. This all plays out to create Fig. 2d–f. It is explained in more detail in Supplementary Note 1.

CO_2_ emissions are another reason (apart from lower costs) to stimulate eTrucks over diesel trucks. Here, our method is based on our earlier method for CO_2_ calculations for electric vehicles^22,32^. Our use of electricity mix over the lifetime has been adopted by BNEF, Tesla and Transport & Environment. We include production emissions of truck, battery, diesel fuel and electricity.

So far, we have considered technological innovation in eTrucks. But we also expect additional organisational innovations by fleet owners, specifically in fleet composition. Based on Basma et al.^5^, we estimate that for a fleet operator, 23% of daily trips could be serviced with an eTruck with a 250 km range, 41% with a 500 km range, and 35% with a 750 km range (leaving 2% for diesel, hydrogen or fuel trucks). Such a fleet would be able to carry higher loads on average and also have a much lower CAPEX. More details are available in Supplementary Note 2.

## Results

### Estimating eTruck performance

**Fig. 3:**
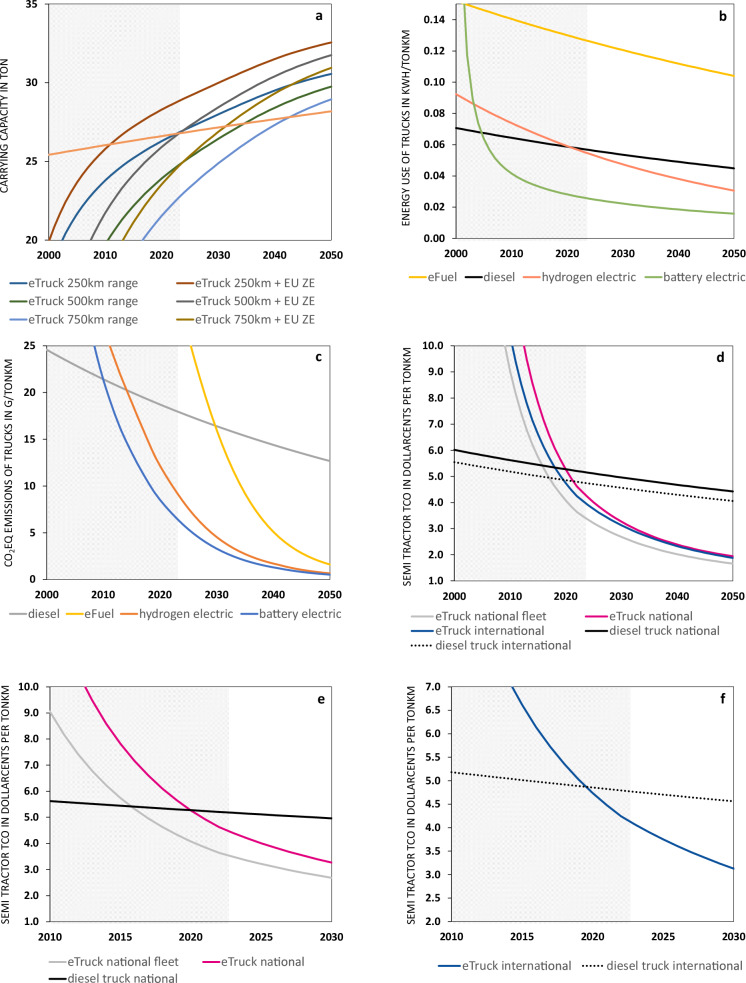
ETruck learning. Over time, eTrucks outperform alternatives on carrying capacity, emissions and price. **a** The cargo carrying capacity of diesel trucks and eTrucks with different battery sizes. **b** The energy use of trucks using eFuel, diesel, hydrogen and electricity. **c** The CO_2_eq emissions of trucks bought in a certain year over their lifetime. **d** The TCO of diesel trucks and eTrucks. **e** Like panel (**d**). but zoomed in and showed trucks driven nationally. **f** Like panel (**d**) but zoomed in and showed trucks driven internationally. (Redesigned eTrucks were not available in the greyed-out areas that indicate the past.).

Figure 3 shows how applying our integral learning curve approach suggests eTrucks could quickly surpass diesel truck performance in terms of cost per ton kilometre (tkm) and even carrying capacity once next-generation eTrucks based on currently available electric vehicle technology become available. We first focus on the key *technological performance* dimensions of weight, energy use and CO_2_ emissions. Then we look at the *economic performance* dimensions of the purchase cost (CAPEX), operation cost (OPEX) and billable load, leading to cost per tkm.

### ETruck weight, energy use and CO_2_ emissions

Weight is of premium importance for a truck because it can carry more (paid) cargo when it weighs less. A typical 40-ton diesel truck can carry up to 27 tons of cargo. The cargo weight is slowly increasing as trucks get lighter (the black line in Fig. 3a). Our model suggests that the cargo weight of eTrucks is increasing much faster than commonly assumed because apart from battery cells becoming lighter, battery packs are also becoming lighter (and they might even become structural, negating their weight) and more importantly the electric drivetrain is increasingly lightweight. The solid red line in Fig. 3a indicates that according to our model, an eTruck with a 750 km range will be able to carry more cargo than a diesel truck by 2037. The dotted red line shows that the EU directive allowing zero-emission trucks to carry 2 tons more brings this tipping point forward to 2029^33,34^. An optimised fleet of eTrucks with 750, 500 and 250 km ranges yields further improvements. The caveat is that eTrucks currently on the road are not yet 2nd generation trucks. Instead they are more akin to retrofits of diesel trucks, adopting redundant parts of diesel drivetrains, and replacing fuel tanks with batteries instead of applying structural batteries. Our analysis suggests further weight reduction is possible once eTrucks become available that were designed to be electric from the ground up.

Energy is the biggest cost component for diesel trucks, and this is where eTrucks gain their biggest advantage. Fig. 3b shows the total energy use per ton of paid cargo per km (tkm) for battery-electric, hydrogen-electric, efuel and diesel trucks, including conversion and transmission losses. The weight reduction mentioned above enables carrying more cargo and causes the initial fast decrease of the energy use per tkm for eTrucks (green line). However, the most important driver for the lower energy use of eTrucks is the much higher efficiency of the electric drivetrain when compared to diesel drivetrains. The hydrogen truck uses more energy than the eTruck because it has to turn electricity into hydrogen and then turn that hydrogen back into electricity. Where an eTruck has 24% total losses (including grid, charging, and drivetrain losses), a hydrogen-electric truck has around 68% losses^35^. (And this is assuming the hydrogen trucks carry the same cargo as diesel trucks, which is slightly optimistic^36^.) Producing eFuel is an energy-intensive process and putting that into a relatively inefficient diesel engine results in the highest energy use of all alternatives^32,35^.

Figure 3c shows the CO_2_ emissions of different drivetrains, with eTrucks having the lowest emissions. In our model, we take energy use and combine that with the CO_2_ emissions of the type of energy used per tkm. To this, we add the production emissions (e.g., of the battery) divided by km driven to arrive at total CO_2_ emission per tkm^22^. For emissions regarding both production and use of the eTruck we have used the average European electricity mix as assumed by the European Environment Agency to which we fitted a logistic curve (2005–2021 *R*^2^ 0.91), roughly going to 112 g/kWh in 2030 and 11 g/kWh in 2050. This means that eTruck production gets cleaner over time and that eTruck emissions are recalculated yearly during the use phase and thus get lower as the eTruck gets older. Importing trucks produced in areas with higher emissions increases emissions somewhat, but the effect is small since most emissions occur during the use phase.

### ETruck CAPEX, OPEX and total cost of ownership (TCO)

Trucking is a highly competitive business, and the total cost of ownership (TCO) per ton km (tkm) is an important performance indicator in the industry. The TCO is determined by the capital expenditure (CAPEX) for the purchase and financing of the vehicle, plus the operational expenditure (OPEX), where energy and maintenance are the biggest cost drivers. We divide the total TCO with the potential cargo weight to get TCO per tkm.

For long-range trucks, eTrucks will remain more expensive in terms of CAPEX up until 2045 in our model, but the size of the disadvantage is quickly decreasing. The most significant decrease in eTruck CAPEX comes from learning in battery cell technology but we can also expect a reduction in battery pack and drivetrain cost. Furthermore, a fleet with eTrucks having a mixed range reduces the CAPEX disadvantage further because shorter-range eTrucks have a lower CAPEX.

OPEX is where the eTrucks have a big advantage as they use only 45% of the energy per tkm compared to a 2022 diesel truck^37^ (see Fig. 3b) while the price of diesel and electricity per kWh is similar. As an example, assuming a lifetime of 1 million km and a diesel price excluding VAT of $1/liter, a diesel truck owner will pay $280k for diesel over the lifetime of the truck. That is twice as much as the sticker price of the diesel truck. (This approximates the situation in Europe. In the US, the taxes on diesel are lower. Still, even in the US, the cost of diesel over the lifetime will be higher than the CAPEX of the truck.)

To get TCO per tkm we take the weight of the truck into account. The eTruck weight decreases over time as battery cells get lighter. However, one should not forget to include the weight of the battery pack, and the much lighter electric drivetrain, as these have a considerable impact, especially once we move to next-generation eTrucks that were redesigned from the ground up to be electric.

Figure 3d shows our model expects the TCO per tkm of eTrucks to become decisively lower once next- generation eTrucks become available. For diesel trucks, we assume the diesel price will rise slightly, but we assume no extra taxation or other policy that impacts the diesel business case. For eTrucks, we distinguish between trucks that are driven nationally and internationally.

Figure 3e zooms in on the TCO of the 65–75% of heavy trucks that only make national trips. These usually return to their ‘home’ depot at night. This means the fleet owner could buy an overnight charger to charge the truck. This option also provides energy at a lower price per kWh than fast charging. The dotted line shows what happens when a fleet owner can combine trucks with different ranges to reduce the CAPEX cost further.

Figure 3f zooms in on the TCO of international trucks. We estimate this to be just 25–35% of all heavy trucks, but they drive almost 50% of kilometres^38–41^. We assume that this international eTruck has to get 30% of its energy from expensive fast charging at rest stops. Recharging can be done in the mandatory 45-min pause after 4.5 h of driving, which is digitally monitored and strictly enforced in the EU. Charging is more expensive for international trucks, but these trucks drive more which helps to earn back the CAPEX cost of the battery. A TCO tipping point could be reached if trucks with a range of 750 km were available for a sticker price of $430k (vs $140k for the diesel truck). In 2030, the total TCO of eTrucks will be half of that of a diesel truck, according to our model. However, this assumes that fast charging and rest-stop charging will be available all over Europe, while there is currently no eTruck charging. It is one of the things policy could address cost-effectively.

## Discussion

The rapid adoption of eTrucks can quickly reduce CO_2_ emissions. Technological learning is making eTrucks increasingly economically competitive by reducing the cost and weight of eTrucks. This is mainly the result of electric car R&D regarding battery cells, battery packs and drivetrain technology. Application to eTrucks would cause tipping points in terms of cost per ton kilometre and carrying capacity. This analysis calculates results for Europe, where high diesel prices and a zero-emission weight allowance bring TCO tipping points forward, but the trend also holds for the rest of the world.

Unfortunately, underestimation of eTruck potential leads to policy uncertainty which negatively affects investment and innovation towards next-generation eTrucks and infrastructure. Furthermore, long-range trucks (that emit over 50% of CO_2_) depend on a fast-charging infrastructure for eTrucks that is lacking. Such an infrastructure would not only need to accommodate fast charging for trucks but also to adopt open standards and foster interoperability. Policies that seek to bring the tipping points forward could either target the eTrucks themselves or the charging infrastructure they need.

Financial stimuli per eTruck should ideally be high in the beginning and come down over time in a predictable manner. Zero emission zones, mandates and other regulations should ideally become more strict over time in a predictive manner. Our model shows policies could accelerate tipping if they reward the use of zero-emission drivetrains, longer electric range, and higher charging speed. Policymakers could also steer battery development in a societally advantageous direction by rewarding more eco-friendly batteries. Policy instruments (apart from research grants) include sales requirements for OEMs, import tariffs, tax discounts, weight allowances, access to city centres, and road taxes.

Regarding charging, tipping could be accelerated with: open standards (avoiding vendor lock-in); interoperability (including universal payment methods); pricing transparency (that is sorely lacking); universal fast-charging coverage (not only the best spots); fast-charging speeds (with higher rewards for faster charging); and vehicle to grid capabilities (especially for long-stay charging locations such as overnight charging). Policies that could drive this are permits for good charging locations, location-based feed-in tariffs that are high to start and get lower over time, preferred treatment for chargers in areas where grid capacity is limited, and dynamic grid tariffs that reward vehicle-to-grid.

The transition from diesel to electric trucks seems unavoidable, but policies that accelerate tipping could greatly affect the speed and societal benefits of this transition.

## Supplementary information

Supplementary_Information_Heavy_Trucks_Auke_Hoekstra

Supplementary_Data_1

## Data Availability

No datasets were generated or analysed during the current study.

## Code availability

Data used in this study was generated from open-source resources and interviews detailed in the Methods section. Processed datasets will be provided upon request.
