# Supplementary material for: Using learning curves to guide the energy transition with the example of heavy electric trucks
Source: NPJ Sustain Mobil Transp. 2025 Apr 2;2(1):14. doi: 10.1038/s44333-025-00029-5 (PMC11964912; doi:10.1038/s44333-025-00029-5)
Supplement: Supplementary file 1 — Supplementary_Information_Heavy_Trucks_Auke_Hoekstra [file 44333_2025_29_MOESM1_ESM.pdf]

Using learning curves to guide the energy transition: the example of heavy electric trucks
SUPPLEMENTARY INFORMATION

Auke Hoekstra[1], Floor Alkemade[2]

[1]Eindhoven University of Technology, department of Mechanical Engineering and Zenmo simulations
[2]Eindhoven University of Technology, department of Industrial Engineering and Innovation Sciences

This supplement explains the values used in our model and how we have constructed fleet functionality.

Supplementary note 1 Main model parameters

1.1 Truck weight

1.1.1 Tractor and drivetrain weight

The 'tractor excluding drivetrain, frame and battery' describes what the weight of the tractor of the semi-truck without these components. It includes components that are the same for both diesel trucks and eTrucks. The parameters were derived from discussions with experts in the industry (e.g., DAF and VDL) and from the IEA report on the future of trucks.[1]

The tractor frame refers to the 'ladder frame' that gives the tractor its rigidity. In our model we gradually implement a 1000 kg structural battery advantage, because in 3rd generation trucks, the battery packaging *is* the component that provides the structural rigidity, in the same way, it already does in electric vehicles with the skateboard chassis. For this parameter we also considered the literature on structural batteries[2–4] and on cell to pack[5].

The 'diesel tractor: diesel drivetrain weight' describes the weight of all the components that together form the drivetrain of the diesel truck. This encompasses not only the motor, gearbox, driveshaft, differential, et cetera, but also the fuel tank, exhaust treatment, et cetera. The diesel drivetrain is not expected to get lighter over time, since it is a mature technology and manufacturers are weary of investing further with the focus on sustainable transport. Moreover, the exhaust norms are becoming increasingly strict, which adds costs to the exhaust system and motor.

The 'etractor electric drivetrain weight' encompasses everything that makes the wheels of the eTruck turn (so motors, motor controllers, inverters, other electronics, et cetera), but it excludes the battery pack. We determined these values in discussions with our colleagues at the Eindhoven University of Technology, Automotive division.[6,7]

The 'etractor: battery cells excl. pack' are detailed below in the next section.

The values for the 'trailer (cargo space pulled by the tractor)' were also determined by expert consultation, after getting initial values and estimates on possible improvements from the IEA report on the future of trucks.[1]

1.1.2 Etractor: battery cell weight

To extrapolate cell battery weight we used historical data on the gravimetric energy density of leading commercially available EVs through time as compiled by various sources.[8–13] This indicates a strong

linear development (R^2 0.96) with density increasing at approximately 7.36 Wh kg^{-1} y^{-1} (Supplementary Figure 1).

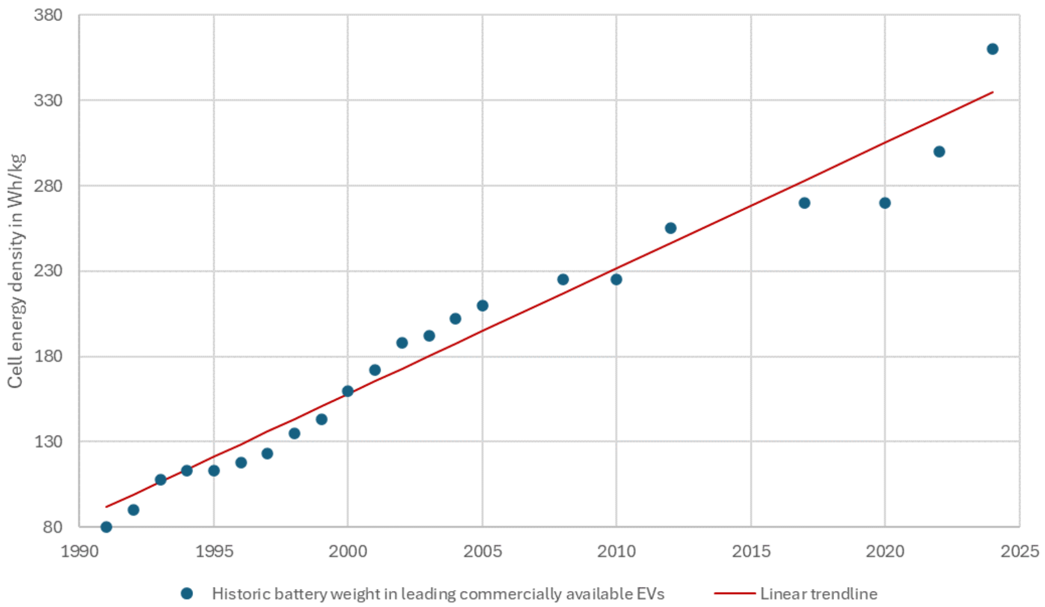

Supplementary Figure 1: Cell density. The cell density of batteries is predictively increasing. Trendline of historical data on the gravimetric energy density of leading commercially available EVs

However, forecasting cell weight development is not straightforward because there are developments on many different fronts. There are developments in the components (e.g. cathode, anode, electrolyte, separator, current collector, casing), the chemistries on another (e.g. the cathode could be LFP, NMC, NCA, LMO, LTO), and the construction methods (e.g. particle size, morphology, synthesis method, doping, compositing, sintering) and many of these developments are interdependent.

Moreover, densities of batteries in the lab have been increasing exponentially at over 7% per year (1995-2024 R^2 0.96)[10]. So far, these weight improvements have often been adopted because lighter batteries require less materials and are therefore cheaper. Lithium-metal, solid-state and Lithium-Sulfur are examples of technologies that promise radically lower weight and cost.

1.2 TRUCK COSTS

In our model, the CAPEX of an eTruck with a 750 km range (which includes financing costs), goes from over $1000k in 2010, to $380k in 2024, $300 in 2030, $220k in 2040, and finally $180 in 2050. Consequently, the model predicts that it will take until 2046 for the the CAPEX of an eTruck with 750km range to become lower than the CAPEX of a diesel truck.

1.2.1 Battery pack cost

In this section, we will construct the learning curve for battery price development as follows. First, we cannot rely on existing or average forecasts. The meta-analysis of Link et al. shows that forecasts are quite heterogeneous, although they show some convergence to the learning curve over time. So, we propose an approach based on this learning curve using Wright's law (based on cumulative production), which yields a 28% cost reduction for every doubling of cumulative production (R^2 0.99). We estimate the cumulative production over time using a conservative but authoritative scenario from the literature (IEA STEPS). Finally, we do a bottom-up sanity check so we know our learning assumptions are reasonable. These steps are described in more detail below.

1.2.1.1 Battery price forecasts become predictively lower over time

Expert predictions of learning regarding renewable technologies (for example, from the IEA and IAMs) are notoriously conservative[14] as shown by Way et al. (e.g. figure 8. And 9.).[15]

When a distinguished but elderly scientist states that something is possible, he is almost certainly right. When he states that something is impossible, he is very probably wrong.
Arthur C Clarke[16]

We examined a meta-study of forecasts by Link et al. in Nature in 2024.[17] Link et al. examined 1100 data points from over 200 unique studies. They conclude that a best fit of datapoints points to a yearly price reduction of 20% over the 2011-23 period, 5.5% from 2024 to 2030 and finally, 3% from 2030 to 2050. The average end result is $175/kWh in 2030, $125/kWh in 2040 and $100/kWh in 2050. The fit is clear but due to the fact that forecasts are very heterogeneous, the R^2 is only 0.49.

However, our analysis of this data shows the forecasts become lower for more recent publication dates. In other words: battery prices are decreasing more rapidly in reality than the forecasters envisioned. We reanalyzed the data of Link et al. hypothesizing that the forecasts would converge to the learning curve. Supplementary Figure 2 shows all forecasts for 2030, 2040 and 2050 (439 data points) ordered by year of publication. The lines show a best-fit curve that conforms to the learning curve we observed in the historical data.

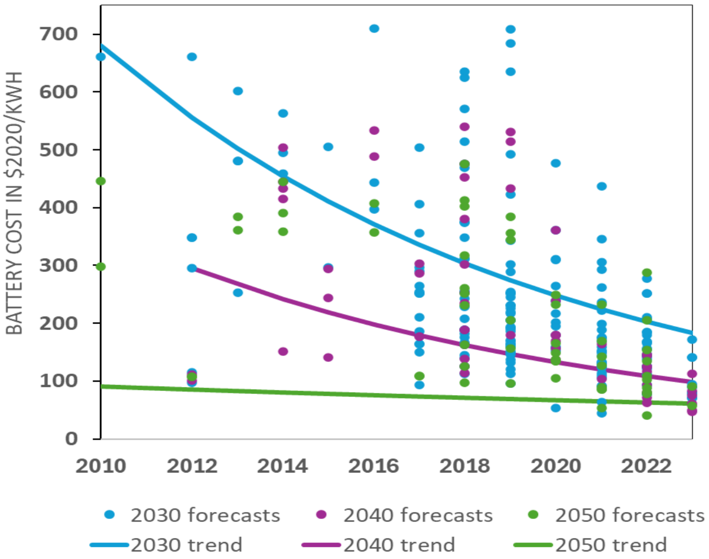

Supplementary Figure 2: Predictions evolve to conform to the historic learning curve. Datapoints from Link et al. Forecasts for 2030, 2040 and 2050 (439 data points) ordered by year of publication. The lines show a best-fit curve that conforms to the learning curve we observed in the historical data. Correlations under the constraint that forecasts will evolve to match historic learning in the target year.

The trendlines show that short-term predictions (for 2030 and 2040) get lower, with, on average, around 9.5% per year, while forecasts by 2050 get lower by 3% per year. This would make them converge on the extrapolation of the historic learning as shown in the next paragraph. Due to the heterogeneity in the forecasts, the fit is far from perfect (R^2 0.45 for 2030, R^2 0.63 for 2040 and R^2 0.44 for 2050, respectively), but nevertheless significant. Moreover, the pattern repeats for 2030, 2040 and 2050, which would be implausible if forecasts would not become predictively lower over time. This provides further support for our hypothesis that extrapolating learning curves based on historical data is a better approach than using expert forecasts.

Further support comes from a comparison of the curve fitted to the forecasts with actual market developments. For 2050 Link et al. find the best-fit points to a price point of $100/kWh, while BNEF already observed prices on the Chinese EV market in April 2024 of $100/kWh for the lightest and most expensive high nickel NMC packs, $80/kWh for average NMC packs, and $75/kWh for LFP packs.[18] (Cell prices are around $25/kWh lower.)

1.2.1.2 Determining the battery pack learning curve using cumulative production

In this article, we propose an approach that follows the data supplied in the form of learning curves. There are roughly two types of learning curves: those that chart how things change over time (Moore's Law is the best-known example) and those that chart how things change for every doubling of production (often called Wright's Law). Usually, Wright's Law fits the data better, and it is also easier to explain theoretically: we learn by doing. Wright's Law also suggests that speeding up the energy transition actually *lowers* the societal cost, because it speeds up learning over time.[15])

To model the effect of learning on eTruck development we first establish how much prices reduce for every doubling of production. In the next step, we will then look at how fast production increases. Finally, we will combine the two to make predictions over time.

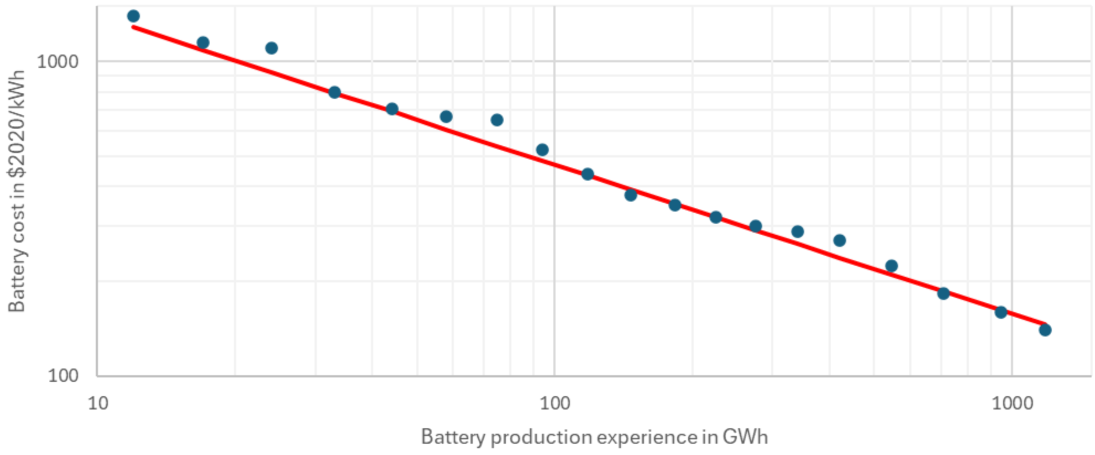

Supplementary Figure 3: Price reductions versus cumulative production of batteries. Datapoints from Way et al.[15] The red line shows a learning curve of 28% per doubling. This learning curve of 28% price reduction for every doubling of cumulative production fits this data well (R^2 0.99).

We use the dataset of Way at all[15] on battery learning (see figure 2 from *their* publication), starting with 10 GWh cumulative production and a price point of $1400/kWh when commercialisation began (around 2010) to 1185 GWh and a price of $140/kWh in 2021. A learning curve of 28% price reduction for every doubling of cumulative production fits this data well (R^2 0.99). The fit for batteries is even better than for solar panels, which are the poster child (called Swanson's Law) for the use of Wright's Law. Determining growth over time and thus learning over time

For the growth of battery production, we use the IEA STEPS scenario.[19] This is the least ambitious IEA scenario, and price reductions would become higher, and the business case of eTrucks would improve faster under a more ambitious NZE scenario, or if historical developments would continue.

STEPS slows down the current growth in battery production of 60% per year (R^2 99% for the period 2016-2023) to 23% from 2024-2030 and 3% from 2030 to 2050. When we combine STEPS with learning of 28% per doubling of cumulative production, the price drops from $151/kWh in 2022 to $42/kWh in 2030 and $16/kWh in 2050.

ETrucks will probably pay a bit more per kWh battery pack than electric cars because the volumes are smaller, which means more overhead in the supply chain. Battery buyers we spoke to in the past years often paid around around 40 to 50% more than the prices on the Chinese market for OEMs as reported by BNEF. These differences will probably become less as the supply chain for eTrucks grows and matures. Our own market research in April 2024 indicated that A-grade LFP cells from CATL cost roughly $80/kWh when bought in small consumer quantities from sellers in the EU while EV OEMs paid around $53/kWh on the Chinese market. This also points to somewhat higher prices in the EU. Finally, battery degradation is more important for eTrucks than for electric cars, and this might create a focus on different types of cells. To account for our focus on eTrucks and the European market, we increased battery pack prices for eTruck OEMs by 50% relative to our prediction for global EV OEMs, a somewhat conservative best estimate of the price difference with EV OEMs.

The resulting function gives an eTruck battery pack price of $150/kWh in 2022, with prices decreasing by 6%/year. The resulting prices are $91/kWh in 2030, $49/kWh in 2040, and $27/kWh in 2050. If we take energy use into account, we estimate pack prices for a 40-ton truck with 750km of range to come down from $500k in 2010, to $100k in 2030 and $27k in 2050 (Supplementary Figure 3).

1.2.2 Powertrain cost

See the explanation in the previous section under 'tractor electric drivetrain weight'. Expert consultation indicates that there are many improvements possible in the electric drivetrain. Theo Hofman, an expert on electric drivetrains, compared the innovation stage for electric drivetrains now to the maturity of combustion drivetrains in the middle of the previous century. E.g. the types of motors, types of magnets, the gearboxes they use (single or e.g. three gears), the number of motors, whether they are located in-wheel, close to the wheel, or in the axle… it all needs further development. Other uncertainties are the type of battery (weight/volume vs power vs cost vs lifetime) or even multiple batteries (one to optimize power, one to optimize density) and, of course, the size of the battery versus the usage pattern versus the charging options.

For diesel trucks, there are very few unknowns and no envisioned step changes. This drivetrain is mostly getting (slowly) more expensive due to stricter demands placed on the exhaust treatment and efficiency.

1.3 Bottom-up sanity check on battery price and weight

Below we discuss whether ongoing innovation developments could realize the price reductions predicted by the learning curve.

Lithium battery pack prices came down from over $1500 per kWh in 2000, with an 8% cost reduction per year for market leaders and 14% for all manufacturers together.[20] For 2022, a price point of $150/kWh is reported.[21,22] For 2030, BNEF predicts $61[23], IHS Markit $73[24], and the Biden administration aims for $60/kWh[25] indicating learning is expected to continue at roughly 8%.. Our modelled price reduction of 6% per year seems within range when looking at 2030, especially if we take into account that 2024 price reductions exceeded expectations.

But what about the long-term model prediction of $27/kWh in 2050? First, we consider the NMC (nickel manganese cobalt) lithium batteries that currently dominate the market, especially for weight-sensitive applications like upmarket long-range electric cars. The price of $150/kWh in 2022 actually represented a slight *increase* because of a temporary supply crunch that temporarily made lithium and cobalt prices ten times higher while nickel became five times more expensive.[26] Contrary to the expectations of many there is now looming oversupply.[27] In the long term, prices are expected to stabilise around $90/kg[28] for lithium, $40/kg for cobalt[29], and $20/kg[30] for nickel. On the battery level,

this would lead to a cost per kWh of $10 for lithium, $10 for cobalt, and $35 for nickel, or $55/kWh for the entire battery with current cell densities. Since the cells were sold at $75 we can infer that some producers manage to make batteries with only a 35% markup on resource prices. Later we will discuss cell weights more extensively, but we emphasize that these prices are for NMC cells that weigh more than 3 kg/kWh, while in the lab we already observe NMC cells that weigh 1.4 kg/kWh,[31] cutting NMC material costs roughly in half (with the lithium amount declining less).

NMC, while popular because it is relatively mature and light, is also one of the most expensive chemistries. And there are many other chemistries that could take over. LFP batteries are the first candidate with commercial maturity and just $12/kWh in raw material cost. At 5 kg/kWh the cells are currently exactly 33% heavier than what we assume in our model for 2024. Using LPF batteries would add 500 kg to our 250 km range truck in 2024 and almost 1500 kg to our 750 km range truck. However, this is less than the advantage currently provided by the European Zero Emission mandate and has a limited impact on the business case, especially for eTrucks with a shorter range. So LPF batteries provide a commercially available option towards cheap batteries. Sodium reduces the material cost to around $3/kWh but is heavier still at 6 kg/kWh. So LFP and sodium are commercially viable and could become much cheaper than we propose but with a roughly 30-50% higher weight than their NMC counterparts. All three chemistries theoretically have room to become roughly two times lighter.

Other options for improvement to the battery itself include lithium metal cathodes, solid-state batteries (with a solid electrolyte) and lithium sulphur (which could get extremely light and cheap). These batteries could all potentially shave both weight and cost off the current designs and some claim to be just a few years away from commercialization. If, e.g., the speed at which LFP and sodium batteries are coming to market and getting lightweight is any indication, some of these options may become available within 5 to 10 years.

Policies could support the adoption of longer-range eTrucks and cheaper and cleaner but heavier batteries, by allowing zero-emission trucks to become heavier. (The maximum axle weight that is critical for road wear and tear could stay the same but the overall weight allowance could increase.) Another option is to stimulate the use of shorter-range trucks that recharge quickly. There have been rapid improvements in the ability of cells to accommodate this faster charging as they get more efficient (and thus less hot) with more precise production and shorter travel paths for ions and electrons. Extremely fast-charging sodium battery cells are a hot topic in research and development.[32,33]

Finally, once batteries reach end of life, they can be recycled[34–36]. This is highly successful in the lab[37–39], but government standards and stimuli would accelerate adoption. At a certain point, mining materials from old batteries would become cheaper than mining it from the earth, allowing closure of battery material mines in the future.

Summarizing, our model learning curves are supported by ongoing innovation trajectories for battery price reduction and weight reduction.

1.4 Battery degradation

The lifetime of a diesel truck is mainly determined by the diesel motor. As the truck ages, the frequency of unplanned maintenance increases. ETruck drivetrains are low maintenance and expected to last longer than the truck, as the drivetrain has few moving parts, the movement is more fluid (not driven by small explosions), and the corroding exhaust is absent. Instead, an eTruck's lifetime depends on the battery's lifetime.

Battery life is even more important for eTrucks than for cars because the battery in eTrucks is cycled more often. Imagine a car with a battery range of 350 km that drives 250 thousand km. It cycles the battery 350 000 / 350 = 715 times. An eTruck with a battery range of 750 km can be expected to drive one million or even 1.5 million km. That requires between 1500 and 2000 charging cycles: two or three times as much.

This is very different from what we saw when electric vehicles took off. The first lithium batteries degraded quickly. E.g., the first air cooled Nissan Leafs had a limited battery life, especially in warmer environments. But Over time, the capacity retention of newer models (Nissan Leaf or otherwise) has improved, as can be observed in the battery degradation comparison tool of GeoTab.[40] One of the biggest fleets is that of Tesla. It reports on battery degradation each year for its (mostly) NMC batteries and consistently sees around 700650 cycles (322 thousand km on the Model 3 and Y with an 85 kWh battery and more on the Model S and Y with a 100 kWh battery) before the battery loses 15% of its capacity.[41] So, these batteries last more than long enough for a car, but not long enough for an eTruck.

However, longer battery life seems possible if we consider current innovation efforts. State-of-the-art cells in 2022 can achieve over 10 thousand cycles.[42,43] Although LFP batteries usually last longer than NMC batteries, there are NMC batteries in the lab that last over 16 thousand cycles,[44] and leading researchers like Jeff Dahn are even discussing four-million-mile batteries.[44–46] Further improvements towards 20 thousand cycles can be expected.[47,48] Promising future developments include next-generation lithium metal batteries that have now achieved ten thousand cycles in the lab.[49]

Another development stimulating R&D in longer cycle life is vehicle-to-grid. New cars are increasingly designed with bidirectional onboard chargers that enable the car battery to provide power to the home, the grid or while camping. Industry experts indicate that the ability to use the car battery appeals to a sense of independence and unlocks lucrative energy and grid capacity trading opportunities. Using the battery this way means the battery should be able to do more cycles but car manufacturers seem increasingly convinced their new cells and better understanding of how to avoid degradation will make this unproblematic.

1.5 ENERGY USE

The values for diesel trucks are well known.[1] Data on eTrucks is now also becoming available.[50] One 3rd generation truck (the Tesla Semi) uses around 1.1 kWh/km with a path to 0.9 kWh/km.

1.6 EMISSIONS

To calculate emissions we followed our earlier approach.[50–52] The biggest unknown is the source intensity of the electricity. Here using average energy intensity is the most appropriate approach (more than arbitrarily determining eTrucks receive marginal emissions), unless one uses a realistic detailed grid model that includes the moment the eTruck is charging (mostly during the night when wind is relatively abundant). To determine emissions of the electricity system in Europe we used values of the European Environment Agency.[53] This data shows a linear reduction of around 7.4 gCO2eq/kWh (1990-2022 R^2 0.97). However, emissions cannot go below zero, so linear extrapolation is not a believable longterm dynamic. A curve that fits both the data (2005-2021 R^2 0.91) and the plans of the EU is a logistic curve. This gives emissions of 112 g/kWh in 2030, 38 g/kWh in 2040, and 11 g/kWh in 2050.

1.7 ENERGY COSTS

1.7.1 Diesel prices excl. VAT

Diesel prices are highly uncertain. Supplementary Figure 4 shows the range of oil price scenarios used by the IEA.[54]

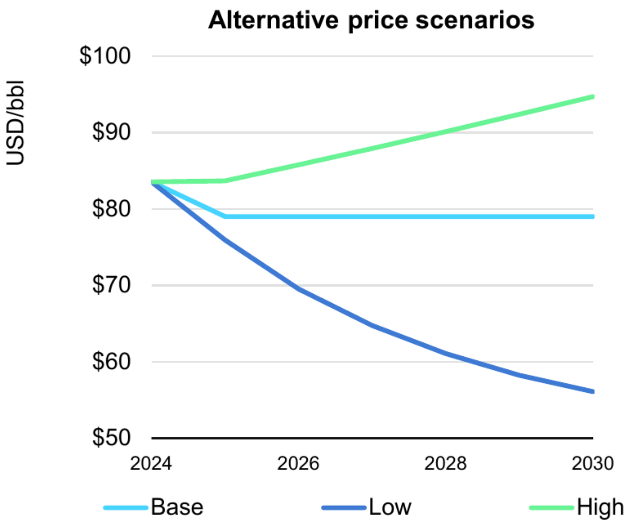

Supplementary Figure 4. Oil prices. Scenarios for oil future oil prices per barrel. Data from the International Energy Agency.

European diesel prices have fluctuated with no discernable trend. See Supplementary Figure 5).

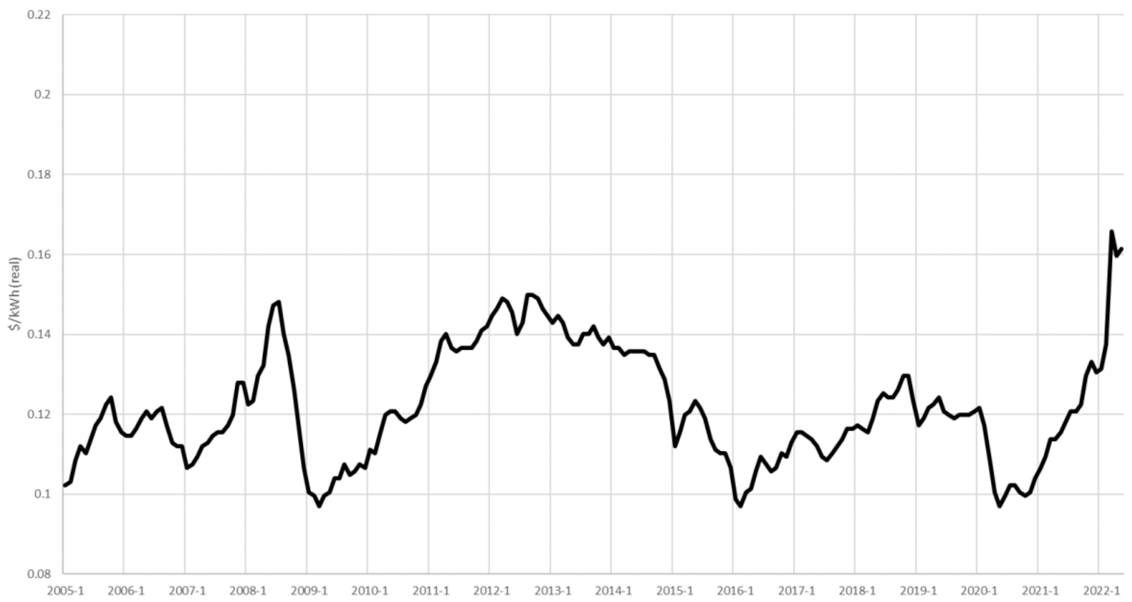

Supplementary Figure 5. Diesel prices. Diesel price developments in Europe ($/kWh adjusted). European diesel prices have fluctuated with no discernable trend.

In the model, we, therefore, use the average diesel price over this period ($1.19/l or $0.122/kWh) and keep it constant after that. This is a conservative estimate if only because volatility is a business risk, and in business, risks are costly (Supplementary Figure 5).

1.7.2 Electricity prices

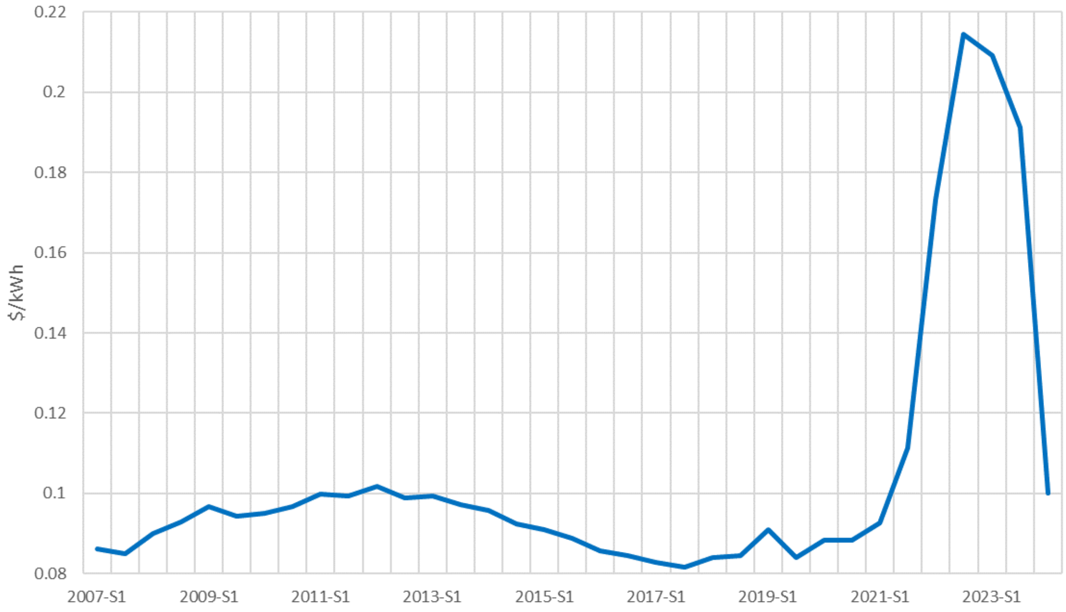

Supplementary Figure 6. Electricity prices. Electricity price developments in Europe ($/kWh adjusted). Electricity prices were relatively stable with a sudden spike that started in 2021

Electricity prices were relatively stable, with a sudden spike that started in 2021 (Supplementary Figure 6). It is commonly thought that initially this was caused by an increase in economic activity post-pandemic. What followed was an increase in gas prices (up to five times higher), due to LNG being redirected to Asia post-pandemic. Since gas is a major component of EU electricity prices, electricity prices soared too. Finally, the invasion of Ukraine by Russia on February 24, 2022, led to the EU seeking new sources of gas (often imported LNG) at high prices.[55] However, prices are returning to pre-pandemic values now, and this whole episode strengthened the resolve of the EU to quickly become less dependent on natural gas for electricity by installing more solar, wind and (recently) batteries. Similar to our estimates on diesel prices, our model uses a price of $0.1/kWh (on the high side compared to the period pre-spike) and keeps this constant over time.

1.7.3 Smart charging advantage

Smart charging can reduce electricity costs if the eTruck is charged in a way that takes energy prices and grid congestion into account. We included this in the model as a 20% price advantage that increases by 1% per year (so after 10 years, it becomes 22%).

1.7.4 Depot charging surcharge

We assume that arranging for a charge point at the depot where the eTruck stays during the night (for trucks that return “home” after the daily drive) costs around $0.05/kWh and that costs reduce by around 3% per year.

We assume charger replacement after 15 years. Assuming average mileage of 115k km per year for non-international trucks multiplied by the average energy use per km over the 15 years of 1.2 kWh/km; the ChargePoint delivers a bit over 2 GWh over that period. The surcharge of $0.05/kWh leads to chargepoint costs of around 2 GWh x 0.05 kWh = $103500 or a little bit over 100k. Most calculations assume a bigger surcharge[56] but since the price of 100kW chargers is already dipping below $25k, we think higher surcharges are unrealistic.

1.7.5 Rest stop charging surcharge

The calculation is similar to what we described for depot charging, but the charger use is far less certain for the entrepreneur installing the charge point and the costs per m2 might be higher. However, since a chargepoint costing around $25k (and becoming cheaper quickly) could yield 400k over a 15 year period if utilized fully, we estimate a surcharge of $0.20 kWh^{-1} in 2022, and decreasing by 2% per year after that.

1.7.6 Fast charging surcharge

This is also uncertain. Tariffs for electric vehicle fast charging are not transparent. From a consumer perspective guidelines on the transparency of tariffs would be beneficial. Fastned, one of the bigger charge point operators is transparent, charging around $0.52/kWh for regular users. That is roughly the same as our base cost of $0.10 plus a $0.40 surcharge in 2022.

As an example, assume an eTruck charge point can deliver 1.5 MW of power, and that a truck charges 70% of that for 30 minutes to charge 500 kWh. Further, assume that a charge point is used 15 times a day (just 4.5 hours out of 24 hours). Then, over a 10-year period that chargepoint would bring in $0.4 x 365 x 10 x 15 x 500 = $ 10 million. And prices for chargepoints (especially their most expensive component the inverter) are strongly and continuously decreasing. We expect that 1.5 MW chargers will soon cost less than $250k to buy. Of course, the cost of the charger is only one part of the business case for the entrepreneur, and there will not be 15 eTrucks per day when he starts out. EU policy could really make a difference by providing, e.g., $1/kWh of subsidy to eTruck charge point operators to get the system started and to slowly phase out that subsidy as the market takes over.

SUPPLEMENTARY NOTE 2 ETRUCK FLEETS

Another innovation that we added to our model is a simple form of eTruck fleet optimization, reflecting the additional organisational and business model innovations that of. Only a few trucks actually drive 750 km per day, and hence, having a fleet with eTrucks that can all drive 750 km per day is unnecessarily expensive.

We use the data on representative fleets from an ICCT study by Basma et al.[56] (see Supplementary Figure 7).

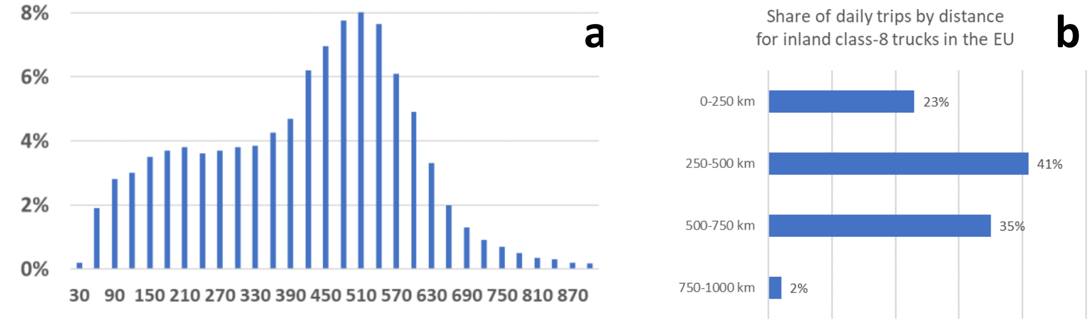

Supplementary Figure 7. Truck driving distance. Share of driving distances per day of trucks in Europe. Based on data from Basma et al.[56] **a)** distribution of driving distances and **b)** share of daily trips by distance.

We used this distribution for our fleet calculations. So a fleet of 100 trucks would have 23 trucks that have a range of 250 km, 41 trucks that have a range of 500 km, and 35 trucks that have a range of 750 km. Apart from that, 2 trucks would be diesel, eFuel or hydrogen trucks (or eTrucks with an even longer range) but we excluded them from our calculations.

First of all, this is relevant for the carrying capacity, as shown below in Supplementary Figure 8. As the graph shows it will take a while before eTrucks with a 750 km range will be lighter than diesel trucks (although with the EU extra weight allowance and a tractor with two pulling axles, they are close). But a truck with a 250 km range is much lighter. And although most cargo is volume-constrained – not weight-constrained – this is something to take into consideration. But most of all, it is relevant for the business case since a smaller battery is cheaper. That is why the fleet shows a better business case than a 750 km truck.

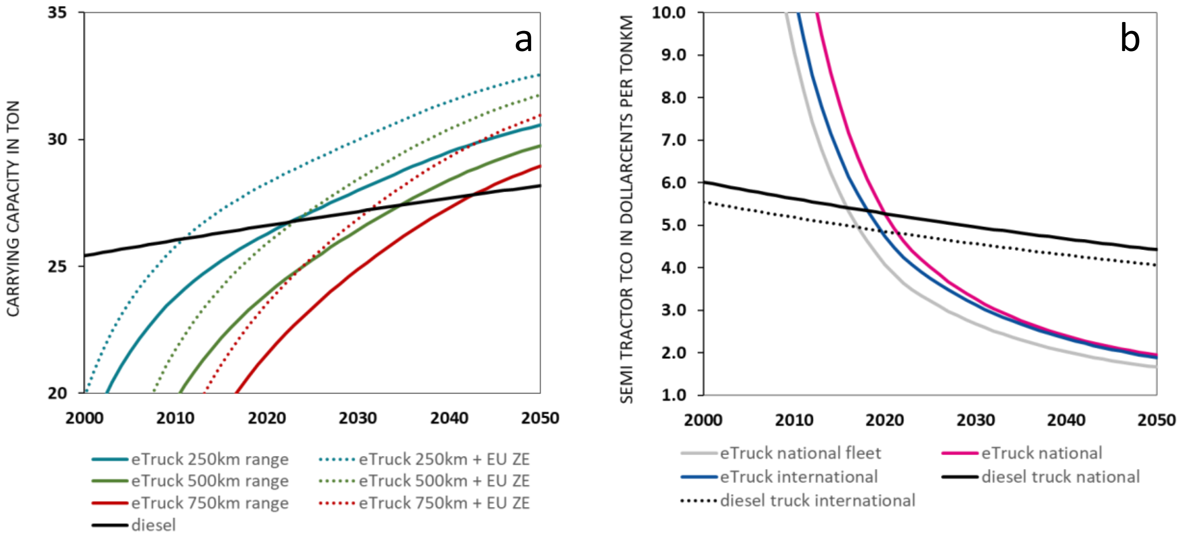

Supplementary Figure 8. Truck weight. The influence of truck weight on the business case. Estimated development of **a.** carrying capacity and **b.** TCO for different trucks.

However, supplementary figure 8 also shows that there is a kind of a catch here, as evidenced by the line designating the international eTruck. The international eTruck pays more for charging because it mainly uses fast chargers. How can it have a better business case than the eTruck with a 750 km range that is used nationally?

The reason is that the international eTruck with a 750 km or range is driven more per day than a national fleet of eTrucks with a 750 km or range. So a bigger battery is actually good for your business case, as long as you manage to make good use of that battery capacity.

Learning curves and a wide range of relevant technologies is a first step. Apart from further researching the learning curves themselves, our approach could be integrated into digital twins using agent-based models.[57] Such models could mimic the actual energy grid (to determine energy pricing, emissions, smart charging advantages, and grid congestion), realistic charger placement, actual travel patterns, and available eTruck models from OEMs. These agent-based models would not only produce more realistic predictions for practical situations but also enable optimal charger placement along highways and give fleet owners and eTruck manufacturers 'hard evidence' to back up (more) investments. Ideally, such a model should make the learning endogenous, which we surmise would show that investing faster would speed up learning and reduce the cost of the transition, as suggested by Way et al.[58] A further improvement would be to add more financial calculations, especially the depreciation of the first owner.

Supplementary References

1. The Future of Trucks – Analysis. *IEA* https://www.iea.org/reports/the-future-of-trucks.
2. Stéphane Melançon. Structural Batteries: The Cars of the Future Are Glued. *Laserax* https://www.laserax.com/blog/structural-batteries (2022).
3. Bilal Akgunduz. What is Structural Battery, CTC/CTB? EV Battery Packs Explained | Licarco. *Licarco - Latest Electric Cars, E-Motors, Vehicle Comparisons* https://api.dolubatarya.com/en/what-is-structural-battery-ctc-ctb-ev-battery-packs-explained/ (2022).
4. Johannisson, W., Zenkert, D. & Lindbergh, G. Model of a structural battery and its potential for system level mass savings. *Multifunct. Mater.* **2**, 035002 (2019).
5. Pampel, F., Pischinger, S. & Teuber, M. A systematic comparison of the packing density of battery cell-to-pack concepts at different degrees of implementation. *Results in Engineering* **13**, 100310 (2022).

6. Verbruggen, F. J. R., Hoekstra, A. & Hofman, T. Evaluation of the state-of-the-art of full-electric medium and heavy-duty trucks: 31st International Electric Vehicle Symposium and Exhibition (EVS 2018) and International Electric Vehicle Technology Conference 2018, (EVTeC 2018). *31st International Electric Vehicle Symposium and Exhibition, EVS 2018 and International Electric Vehicle Technology Conference 2018, EVTeC 2018* (2018).

7. Verbruggen, F. J. R., Silvas, E. & Hofman, T. Electric Powertrain Topology Analysis and Design for Heavy-Duty Trucks. *Energies* **13**, 2434 (2020).

8. Yang, X.-G., Liu, T. & Wang, C.-Y. Thermally modulated lithium iron phosphate batteries for mass-market electric vehicles. *Nat Energy* **6**, 176–185 (2021).

9. Placke, T., Schmuch, R., Dühnen, S. & Winter, M. Lithium-Ion, Lithium Metal and Alternative Rechargeable Battery Technologies: The Odyssey for High Energy Density. *Journal of Solid State Electrochemistry* **DOI: 10.1007/s10008-017-3610-7**, (2017).

10. Li, Q., Yang, Y., Yu, X. & Li, H. A 700 W·h·kg−1 Rechargeable Pouch Type Lithium Battery. *Chinese Phys. Lett.* **40**, 048201 (2023).

11. Lima, P. CATL expects to introduce NCM 811 battery cells next year - 🔋PushEVs. https://pushevs.com/2018/08/14/catl-expects-to-introduce-ncm-811-battery-cells-next-year/ (2018).

12. Xu, J. *et al.* High-Energy Lithium-Ion Batteries: Recent Progress and a Promising Future in Applications. *ENERGY & ENVIRONMENTAL MATERIALS* **6**, e12450 (2023).

13. Zu, C.-X. & Li, H. Thermodynamic analysis on energy densities of batteries. *Energy & Environmental Science* **4**, 2614–2624 (2011).

14. Hoekstra, A. E., Steinbuch, M. & Verbong, G. P. J. Creating Agent-Based Energy Transition Management Models That Can Uncover Profitable Pathways to Climate Change Mitigation. *Complexity* (2017) doi:10.1155/2017/1967645.

15. Way, R., Ives, M. C., Mealy, P. & Farmer, J. D. Empirically grounded technology forecasts and the energy transition. *Joule* **6**, 2057–2082 (2022).

16. Clarke's three laws. *Wikipedia* (2024).

17. Link, S., Stephan, A., Speth, D. & Plötz, P. Rapidly declining costs of truck batteries and fuel cells enable large-scale road freight electrification. *Nat Energy* 1–8 (2024) doi:10.1038/s41560-024-01531-9.

18. EVO Report 2024 | BloombergNEF | Bloomberg Finance LP. *BloombergNEF* https://professional.edit.cirrus.bloomberg.com/bnef/electric-vehicle-outlook/.

19. IEA. *Batteries and Secure Energy Transitions – Analysis*. https://www.iea.org/reports/batteries-and-secure-energy-transitions (2024).

20. Nykvist, B. & Nilsson, M. Rapidly falling costs of battery packs for electric vehicles. *Nature Climate Change* **5**, 329–332 (2015).

21. Batteries For Electric Cars Speed Toward a Tipping Point. *Bloomberg.com* (2020).

22. Lithium-ion Battery Pack Prices Rise for First Time to an Average of $151/kWh. *BloombergNEF* https://about.bnef.com/blog/lithium-ion-battery-pack-prices-rise-for-first-time-to-an-average-of-151-kwh/ (2022).

23. Colin McKerracher. EVO Report 2021 | BloombergNEF | Bloomberg Finance LP. *BloombergNEF* https://about.bnef.com/electric-vehicle-outlook/.

24. Nicholas Yiu *et al. The Battery Report 2021*. https://medium.com/batterybits/the-battery-report-2021-442ed2a06324 (2022).

25. National Blueprint for Lithium Batteries. *Energy.gov* https://www.energy.gov/eere/vehicles/articles/national-blueprint-lithium-batteries.

26. Battery Price Declines Slow Down in Latest Pricing Survey. *Bloomberg.com* (2021).

27. China Already Makes as Many Batteries as the Entire World Wants. *Bloomberg.com* (2024).

28. Mickleboro, J. Here's the lithium price forecast through to 2025. *The Motley Fool Australia* https://www.fool.com.au/2022/12/08/heres-the-lithium-price-forecast-through-to-2025/ (2022).

29. *Quarterly Cobalt Market Update 2022 Q3*. https://www.cobaltinstitute.org/wp-content/uploads/2022/10/Q3-2022-market-report_Cobalt-Institute_EN.pdf.

30. *Commodity Markets Outlook*. https://openknowledge.worldbank.org/bitstream/handle/10986/38160/CMO-October-2022.pdf (2022).

31. Li, Q., Yu, X., Li, H. & Chen, L. The road towards high-energy-density batteries. *The Innovation Energy* **1**, 100005–4 (2024).

32. Wang, Q. *et al.* Fast-charge high-voltage layered cathodes for sodium-ion batteries. *Nat Sustain* **7**, 338–347 (2024).

33. Usiskin, R. *et al.* Fundamentals, status and promise of sodium-based batteries. *Nat Rev Mater* **6**, 1020–1035 (2021).

34. Baars, J., Domenech, T., Bleischwitz, R., Melin, H. E. & Heidrich, O. Circular economy strategies for electric vehicle batteries reduce reliance on raw materials. *Nat Sustain* **4**, 71–79 (2021).

35. Harper, G. *et al.* Recycling lithium-ion batteries from electric vehicles. *Nature* **575**, 75–86 (2019).

36. Tian, G. *et al.* Recycling of spent Lithium-ion Batteries: A comprehensive review for identification of main challenges and future research trends. *Sustainable Energy Technologies and Assessments* **53**, 102447 (2022).

37. Zhou, Y. *et al.* Comprehensive recovery of NCM cathode materials for spent lithium-ion batteries by microfluidic device. *Separation and Purification Technology* **294**, 121185 (2022).

38. Lithium-Ion Battery Recycling Prize Drives Recovery of Spent Batteries. https://www.nrel.gov/news/program/2021/lithium-ion-battery-recycling-prize-drives-recovery-spent-batteries.html.

39. Batterie-Recycling in Europa (Stand: Februar 2022). *Battery-News.de* https://battery-news.de/index.php/2022/02/18/batterie-recycling-in-europa-stand-februar-2022/ (2022).

40. How Long do Electric Car Batteries Last? What 6,300 EV Batteries Tell Us. *Geotab* https://www.geotab.com/blog/ev-battery-health/.

41. Tesla. *Tesla 2023 Impact Report*. 160 https://www.tesla.com/impact (2023).

42. Eldesoky, A. *et al.* The Role of Long Lifetime Li-Ion Cells in a Sustainable Future. *Meet. Abstr.* **MA2022-02**, 222 (2022).

43. Liu, J. *et al.* The TWh challenge: Next generation batteries for energy storage and electric vehicles. *Next Energy* **1**, 100015 (2023).

44. Aiken, C. P. *et al.* NMC as a Superior Alternative to LFP for Long-Lived Low Voltage Li-Ion Cells. *J. Electrochem. Soc.* **169**, 050512 (2022).

45. Harlow, J. E. *et al.* A Wide Range of Testing Results on an Excellent Lithium-Ion Cell Chemistry to be used as Benchmarks for New Battery Technologies. *J. Electrochem. Soc.* **166**, A3031 (2019).

46. Four-million-mile battery is now a reality. https://nickelinstitute.org/ (2022).

47. Hosen, M., Jaguemont, J., Van Mierlo, J. & Berecibar, M. Battery lifetime prediction and performance assessment of different modeling approaches. *iScience* **24**, 102060 (2021).

48. Batteries 2020 – Lithium-ion battery first and second life ageing, validated battery models, lifetime modelling and ageing assessment of thermal parameters. in.

49. Ye, L. & Li, X. A dynamic stability design strategy for lithium metal solid state batteries. *Nature* **593**, 218–222 (2021).

50. Anne Kleijn *et al. STREAM Freight Transport 2020 - Emissions of Freight Transport Modes*. (2021).

51. Hoekstra, A. The Underestimated Potential of Battery Electric Vehicles to Reduce Emissions. *Joule* **3**, 1412–1414 (2019).

52. Hoekstra, A. & Steinbuch, M. Comparing the lifetime green house gas emissions of electric cars with the emissions of cars using gasoline or diesel. 30.

53. Greenhouse gas emission intensity of electricity generation. *European Environment Agency* https://www.eea.europa.eu/data-and-maps/daviz/co2-emission-intensity-15.
54. IEA. Oil 2024 - analysis and forecast to 2030.
55. How are EU electricity prices formed and why have they soared? *Eurelectric - Powering People* https://www.eurelectric.org/in-detail/electricity_prices_explained/.
56. Basma, H., Saboori, A. & Rodríguez, F. *Total Cost of Ownership for Tractor-Trailers in Europe: Battery Electric versus Diesel*. 49 https://theicct.org/publication/total-cost-of-ownership-for-tractor-trailers-in-europe-battery-electric-versus-diesel/ (2021).
57. Hoekstra, A., Steinbuch, M. & Verbong, G. Creating Agent-Based Energy Transition Management Models That Can Uncover Profitable Pathways to Climate Change Mitigation. *Complexity* **2017**, 1–23 (2017).
58. Way, R., Ives, M. C., Mealy, P. & Farmer, J. D. Empirically grounded technology forecasts and the energy transition. 23.
